# 3d cranial reconstruction using titanium implant – a case report

**DOI:** 10.4314/ahs.v22i3.41

**Published:** 2022-09

**Authors:** Sunantha Selvaraj, Jayachandran Dorairaj, N Shivasankar

**Affiliations:** 1 Vinayaka Mission's Sankarachariyar Dental College, Prosthodontics; 2 Vinayaka Mission's Sankarachariyar Dental College, Periodontics; 3 Confident Dental Laboratory, Director

**Keywords:** Cranial implants, titanium, 3D Printing, Fused deposition modelling Printing, Selective laser melting (SLM) technology

## Abstract

Cranioplasty is a neurosurgical procedure done to cover the defective or deficient skull bone. The cranial reconstruction rejuvenates the patient by protecting and restore intracranial structures and pressure thus improving the esthetic appearance. Thus improves the neurological and psychological wellbeing of the patient. The blending Advancement of computer technology in medical and dental science allowed the 3D reconstruction of several anatomical structures for various medical procedures by designing the custom-made implants.

**Procedure:**

This case report describes the methodology used to design a custom-made cranial implant for a 38-year-old patient who had traumatic injury in the right temporosagital region of the skull caused by a road traffic accident . 3D reconstruction of the cranial vault was done using CAD designing and Selective laser melting (SLM) technology printing.

**Discussion:**

The presicion of the prosthesis was good thereby the surgical time was reduced and eliminates any errors in operating theatre and successfully implanted. The patient's esthetics was restores , allowing the patient to safely perform daily activities with full confidence.

**Conclusion:**

The use of 3D reconstruction techniques in managing exhaustive surgeries aids to reduces the possibility of errors during surgery, precise and passive fit and provides better implant stability. Thus 3D printing technology has boomed its use in various field of medicine.

## Introduction

The reconstruction and rehabilitation of large cranial defects((> 25 cm^2^) is challenging to functionally protect the underlying brain, prevent any infection to the vital structures, esthetic consideration and affordability of the patient^1^,^2^. Various materials were used to reconstruct the cranial defects namely autologous bone, metal or mesh plates, poly-methyl-methacrylate (PMMA), hydroxyapatite ceramics or carbon fiber reinforced polymer. PEEK(Polyether-ether-ketone) polyetherketoneketone (PEKK)and PAEK (polYacryl ether ketone) are the recent trends in the cranioplasty material . Each material poses their own advantages and disadvantages^3^. Cranial Implants made from PMMA are stable, biocompatible, chemically inert, nonconductive, radioluent, and inexpensive and can be easily placed and modified^4^–^6^. Thus it accomplishes the requirements but durability and the reaction of the PMMA implant is questionable and technique sensitive. Titanium implants used for cranioplasty is considered best biocompatible property and it is one of the most widely used biomaterials for calvarial fixation or reconstruction^7^.

## Case Report

A 38-year-old male patient who had traumatic injury in the right tempEro sagital region of the skull caused by a road traffic accident 5 yrs back . Neuro cranial surgery was done and the fractured skull bone removed and titanium mesh was placed. The most common material used is grade 5 surgical titanium (i.e., Ti-6Al-4V), usually referred to as “titanium” or Ti-6-4. Titanium was used in the cranium defects with the fixation devices like plates and screws and mesh.

**Figure 1:**
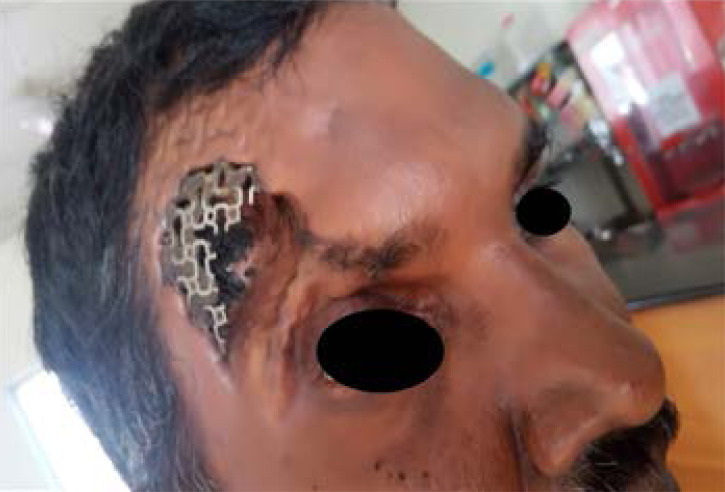
Exposed Titanium Mesh

After a period of 4 yrs the patient had reported to neurosurgeon with implant fenestration through the scalp. The edge of the previously placed titanium mesh was exposed at the pterion region and skin surrounding the defect showed remarkable thinning and fragile (fig-1). Thinning of scalp resulted in reduced vascularity thereby had appearance of scalp ulcerations. So the Ti mesh was surgically removed. As the scalp thickness was very thin the defect area started to sink in and thus intra cranial pressure shooted up. Patient slowly had detoriation in the movement of left hand. Interdisplinary approach of neuro surgeons and prosthodontist a custom made cranial Ti implant was planned.

**Figure 2:**
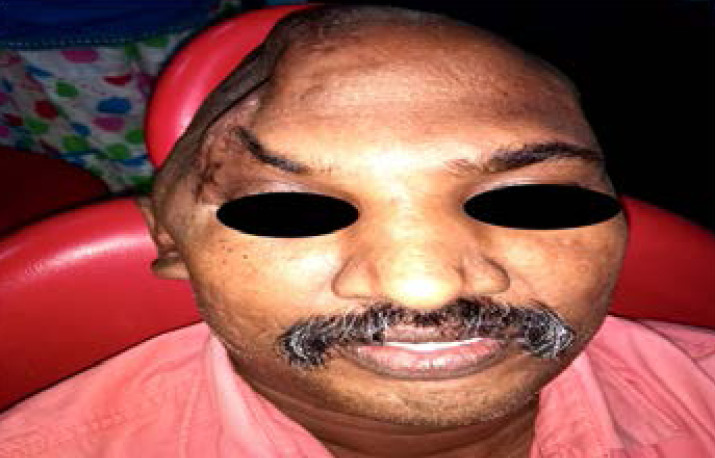
Right Cranial Defect

The patient had a large defect in the left cranial measuring about 18cm x 12cmx 8mm anterio posterior, mesio-lateral and depth respectively (fig-2). Through case history was taken and CT Scanning was adviced. We didn't attempt to take impression of the defect as the skin overlying was very thin.

## Procedure

**Figure 3:**
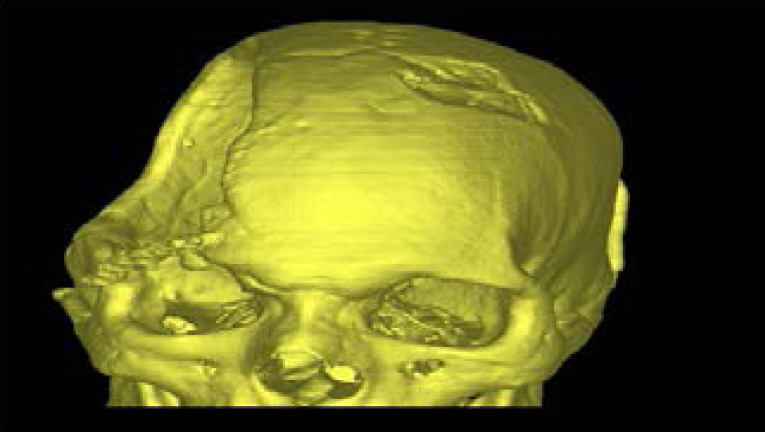
3D virtual image of the skull -frontal view

**Figure 4:**
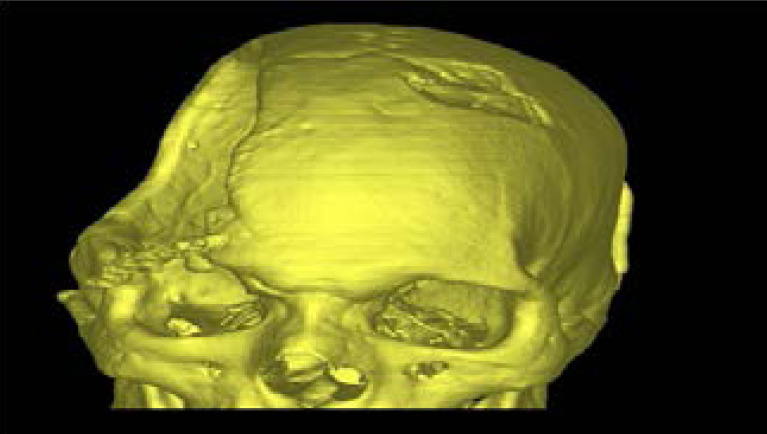
3D virtual image of the skull -proximal view

A high-resolution CT scan of the entire skull is performed on the patient to know the defect area and extent. The acquired images in DICOM(Digital Imaging and Communications in Medicine) format are transferred to the manufacturing company . An accurate 3D virtual image of the skull is created, via software, for the patient (fig.3a &b). Using this 3D virtual images of skull 3D plastic prototype of the patient's skull defect was printed. Virtual 3D Model of the patient's skull obtained from DICOM. Fused deposition modeling, or FDM 3D Printing, is a method of additive manufacturing where plastic filament were squeeze melted and then deposited in layers on the printing .FDM technique was used to build 3-D model of the defective skull vault. Such prototype 3D Model of the skull gives an exact idea to the neuro surgeon the extent of the defect and the thickness of the bone bounderies.(fig 4).

**Figure 5:**
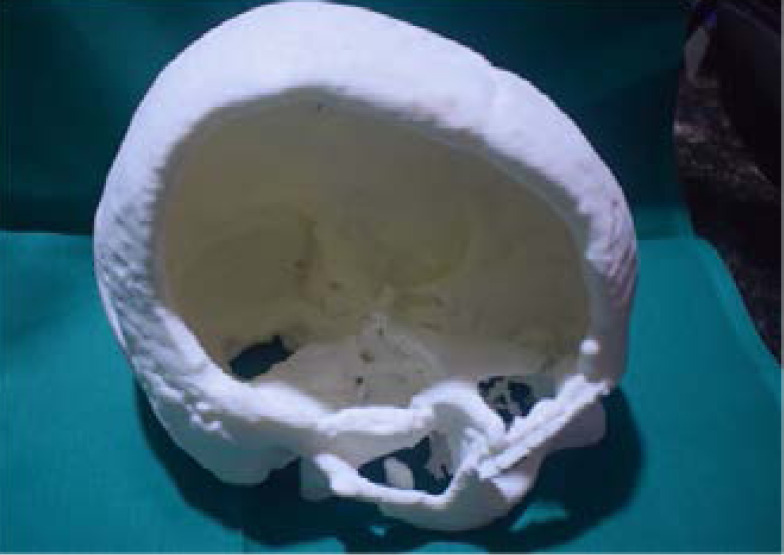
FDM 3D Printed cranial model

**Figure 6:**
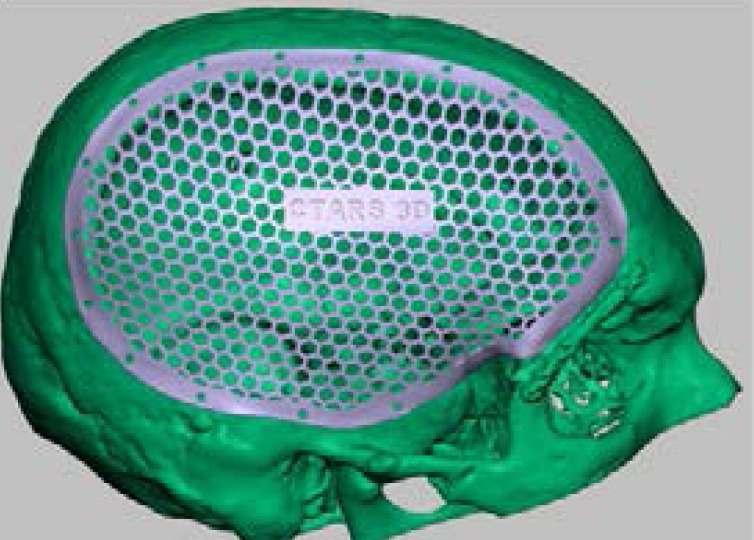
CAD- Resin mesh

**Figure 7:**
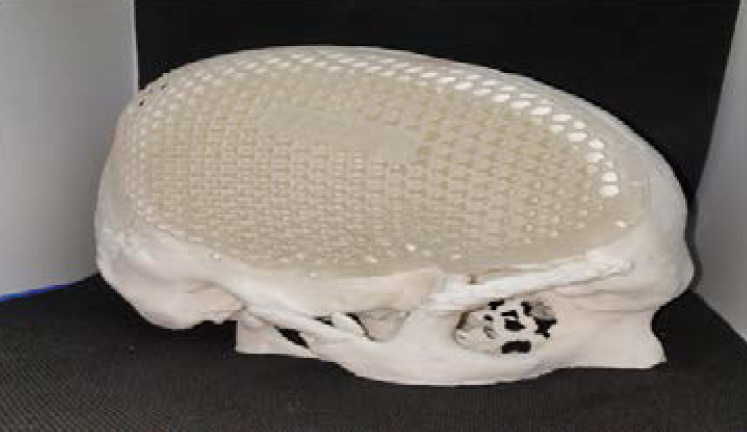
Printed trial implant mesh

Using virtual 3-D model of the defective skull vault was, 3D trial implant mesh was designed (fig.5) and milled using additive technique using Formlabs clear resin.(fig.6). The resin trial implant mesh was inspected thoroughly for the extension, thickness and the diameter of the screw holes. The trial implant was rechecked with the patient's defective area (fig 7).

Once the trial phase is over, the cranioplasty implant grafts was made of titanium grade -5 medical grade, modelled by CAD/CAM technology and produced by Selective laser melting (SLM) technology. SLM is in an additive manufacturing method specially developed for 3D Printing metal alloys. The full melting process allows the metal to form a homogeneous block with good resistance^10^. It creates parts additively by fusing titanium metal powder particles together in a full melting process . This novel technique provides the precise shape of the titanium implant in a virtual 3D model of the patient's skull . With the SLM technique, the previously designed titanium graft was printed in SLM R 125 machine (by SLM Solutions Gmbh , Germany) . The specification of SLM 125machine are 125mmx125mmx125mm (LxBxH) build envelope. 3D optics single IPG fibre laser configuration. Increments can be added in variable layer thickness about 20–75microns

**Figure 8:**
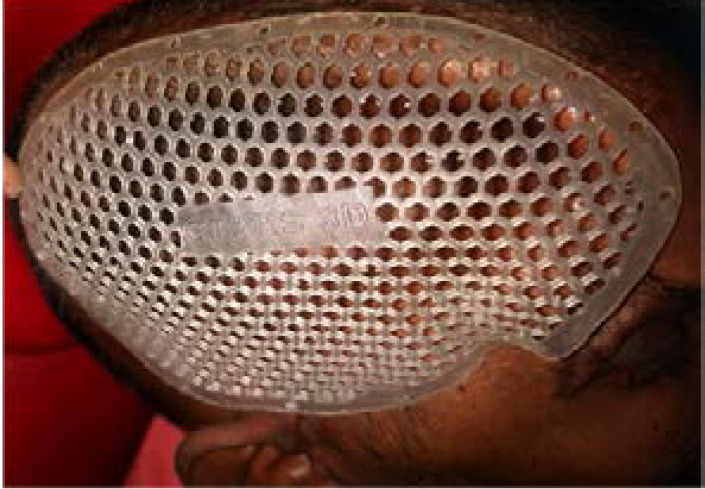
Resin mesh Try in

**Figure 9:**
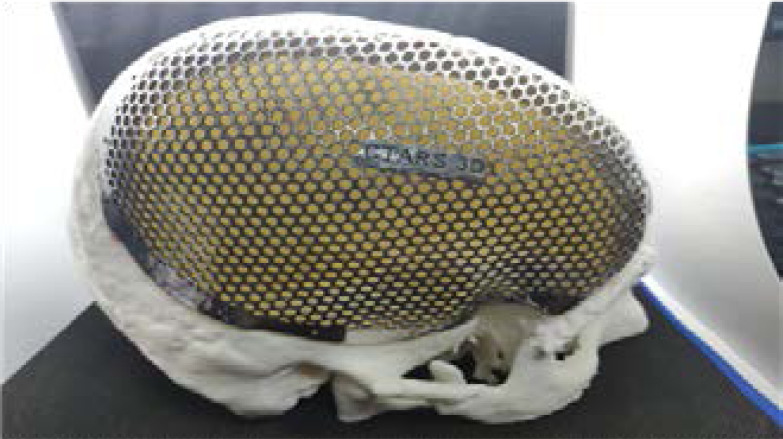
SLM Printed Ti implant

The printed metal implant then tried on the 3D plastic prototype of the skull defect to ensure the best clinical and esthetic results before surgical implantation (Fig. 8). The custom-made SLM titanium cranial implant grafts are provided with holes for drainage and textured surfaces to improve their integration with soft tissue. Moreover, the graft's thin and definitely shaped margins precisely follow the bone defect margins. The fixation is performed with titanium screws directly onto the bone circumference(fig 9). Thin portions of the scalp with a poor vascularity were all removed. The scalp tissue was mainly closed using a primary closure technique, though scalp reconstruction was performed with a rotation flap in cases of large scalp ulceration (> 5 cm^2^). A postoperative CT scan was dine to evaluate the surgical outcome.

## Discussion

The primary goals of cranial vault reconstruction are immediate cerebral protection, re-establishment of intracranial domain, healthy soft tissue coverage, and adequate cosmetics. The ideal quality of a material for cranioplasty are that easy and complete coverage of the cranial defect, which are radiotransparent, low infection rate^11^, biocompatibility, good elastic modulus and resistance to strain, and the maintenance of its chemical and physical properties over time^1^, ^3^. The advantage of Ti alloy is that it possess most of the properties of alloplastic bone substitutes in cranioplasty. When compared to PMMA, Ti mesh, 3D Ti cranial implant is in higher side .Proper treatment planning and advancement in the technology has made the fabrication easier. Considering the size of the defect and cost , PEEK material was not used^12^ in this case.

The surgical time was 80% shorter than that for the same type of surgery in which standard commercial implants titanium meshes were used. Eventually the error and complication during the surgical procedures is also been reduced due to the accuracy of the customised cranial implant fabricated . Finally the appearance of the patient was restored, allowing the patient to safely perform daily activities.

**Figure 10:**
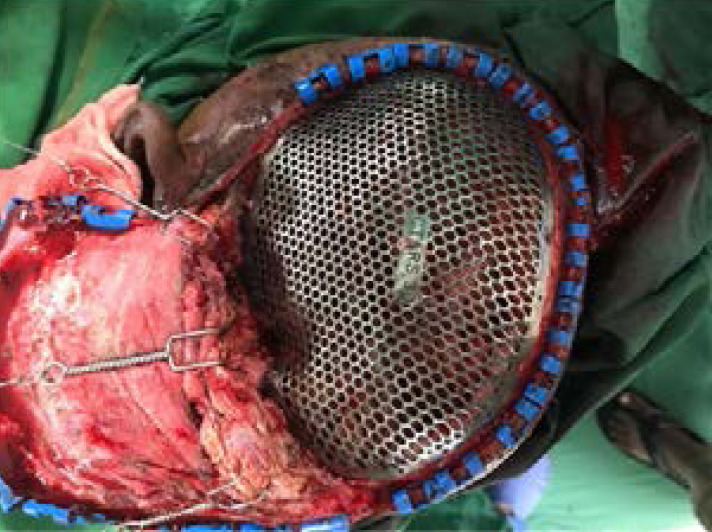
Ti graft implantation

During the first postoperative week(fig 10), the patient reciprocated well in his level of alertness, orientation, speech, motor skills, and social interaction. He was able respond well to simple commands and regognize his family members. At the time of discharge (i.e., 4 weeks after surgery), he had subdural abscess as scalp reconstruction was done by rotational flap .Antibiotic therapy was given and he was in his regular diet and ambulate with assistance. Between 6 months to 1 year postoperatively, his incisions healed completely, and CT scan showed no recurrence of subdural abscess. He demonstrated significant progress with regard to speech, independent ambulation, and ability to engage in activities of daily living. Despite residual difficulty with word recall and short-term memory at 1 year follow up, the patient was very satisfied with his functional status and physical appearance.

## Conclusion

In a large cranial defects the abnormal intracranial pressure in patients can be improvised by proper cranioplasty thereby normalizing cerebral hydrodynamics^13^. The present report demonstrates the efficacy of delayed cranial vault reconstruction using precise titanium cranial implant using CADCAM technology over the commercially available Ti mesh. He had a excellent functional recovery and no evidence of recurrent infection on long-term follow-up.

## Figures and Tables

**Figure 11:**
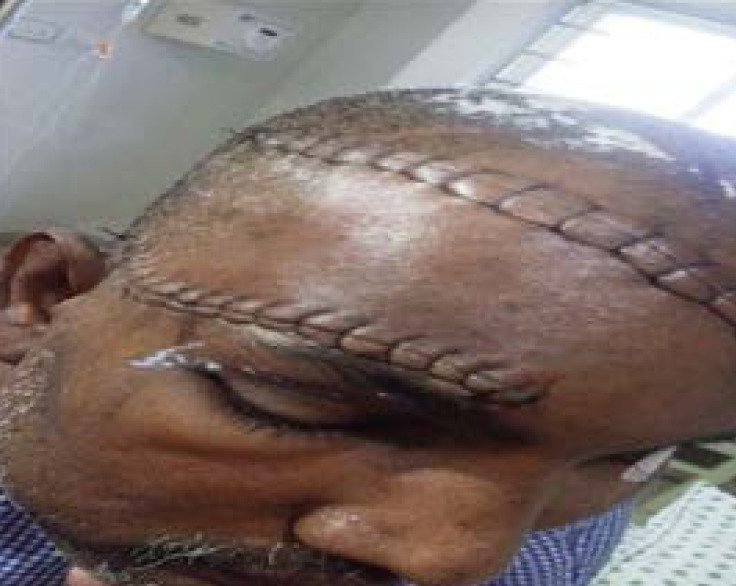
Post operative
